# In Vitro Antioxidant and Antidiabetic Effects of Atlantic Mackerel and Sardine By-Product Hydrolysates

**DOI:** 10.3390/md23100393

**Published:** 2025-10-04

**Authors:** Cristina Fuentes, Samuel Verdú, Raúl Grau, José Manuel Barat, Ana Fuentes

**Affiliations:** University Institute of Food Engineering—FoodUPV, Universitat Politècnica de València, Camino de Vera s/n, 46022 Valencia, Spain; saveram@upvnet.upv.es (S.V.); rgraume@tal.upv.es (R.G.); jmbarat@tal.upv.es (J.M.B.); anfuelo@upvnet.upv.es (A.F.)

**Keywords:** fish waste, fish by-products, hydrolysates, enzymatic hydrolysis, peptides, bioactivity

## Abstract

This work evaluates the effect of raw material and protease enzymes on the antioxidant and antidiabetic potential of fish by-product hydrolysates. For this, mackerel (*Scomber scombrus*) and sardine (*Sardina pilchardus*) by-products were hydrolyzed using papain, pepsin, and Protamex^TM^. Pepsine produced hydrolysates with a lower degree of hydrolysis (34%) and longer peptide chain lengths (2.9), regardless of the raw material. The highest DH was found for the sardine by-products hydrolyzed with papain and Protamex^TM^, exceeding 55% for both enzymes. The mackerel by-product hydrolysates exhibited higher antioxidant activity, while the sardine samples showed more potent antidiabetic effects. Accordingly, sardine by-products and pepsin would be preferable for producing hydrolysates with antidiabetic potential, and mackerel by-products, hydrolyzed papain, and Protamex^TM^ would be useful for producing antioxidant peptides. This study demonstrates the potential of Atlantic mackerel and sardine waste as a source of bioactive peptides and the opportunity for revalorizing these by-products.

## 1. Introduction

Global fish production has reached a record high and is expected to continue increasing due to population growth and consumers’ awareness of its high nutritional value [1]. Small pelagic species, including Atlantic mackerel (*Scomber scombrus*), and sardine (*Sardina pilchardus*), entailed 30% of the total volume of all fishery and aquaculture products produced in the EU in 2020, with 1.23 million tonnes [2]. Together with herring, Atlantic mackerel (*Scomber scombrus*) and sardine (*Sardina pilchardus*) are the most consumed small pelagics [2] and after tuna, sardine and mackerel are the main canned fish species produced worldwide [3]. These species are well appreciated for their high protein, omega-3 fatty acids, vitamins, calcium, and other nutrient content, but low mercury concentration [4,5].

The expansion of fisheries and aquaculture production will increase fish waste volumes. The FAO estimates that by-products may constitute up to 70% of processed fish depending on species, fish size, and processing type [1]. The waste generated while processing mackerel and sardines is usually discarded or employed to obtain low-value by-products, such as fishmeal and fish oil [1]. However, fish waste like viscera, frames, skins, tails, or fins, may also be used as a source of high-value compounds to develop new products or ingredients for food, biotechnological, and pharmaceutical applications [6,7]. Moreover, fish by-product valorization is a pressing demand for sustainable waste management strategies and for implementing the SDG 2030 goal of “zero waste” [8]. Notable initiatives have been implemented in different countries to valorize fish by-products and achieve “zero discards”. These initiatives include measures to eliminate fish discards or reduce fishery waste by fully utilizing fish by-products and providing added value to these residues. All these initiatives have been included in global policies worldwide with the aim of transforming fishery waste into value-added resources through sustainable technologies [1,8].

Fish waste has a similar composition to that of fish filets intended for human consumption [6,9]. In addition to other components such as minerals, astaxanthin, chitin, or essential fatty acids, fish by-products exhibit a high protein content [9,10]. Hence, bioactive peptide extraction provides an exceptional tool for fish waste valorization [7].

Several studies have described the functional and bioactive properties of protein hydrolysates obtained from fish and fish by-products, including antimicrobial, antihypertensive, antioxidant, or antidiabetic benefits [11,12,13,14,15]. Protein hydrolysates’ bioactivity has been correlated with the size of their peptide fragments and amino acid composition [16]. Indeed, the bioactive activity of these peptides has been related to their high content of hydrophobic amino acids, such as alanine, leucine, isoleucine, methionine, phenylalanine, proline, tyrosine, tryptophan, or valine, that facilitate peptide–cell membrane interactions and promote their biological effects [17]. Furthermore, hydrolysate properties, including peptide size and amino acid composition, are affected by different hydrolysis process parameters, such as pH, temperature, enzyme type, and enzyme concentration, among others [11,14,18,19,20]. In this context, this work aimed to study the in vitro antioxidant and antidiabetic potential of fish by-product hydrolysates by analyzing the influence of raw material and the used enzyme on their degree of hydrolysis and their bioactive properties.

## 2. Results

### 2.1. Proteolysis Analysis

The effect of fish species and enzyme type on the by-product proteolysis was evaluated by determining the total protein content, total soluble protein, TCA-soluble peptides, free amino groups, DH, and PCL (Table 1). The total protein content of fish hydrolysates ranged from 45% to 58%. The soluble protein content was higher for mackerel than for sardine by-product hydrolysates. The total soluble proteins content for the mackerel samples ranged from 2.92 mg/g to 3.19 mg/g, while the highest value was 2.83 mg/g for sardine, corresponding to the sardine by-products hydrolyzed by pepsin (SE). For the TCA-soluble peptides, the highest concentration was for SR (145.41 mg/g), followed by MA (141.92 mg/g) and SA (138.83 mg/g), while a smaller amount was found for ME (64.65 mg/g). The higher free amino group content was observed in the sardine by-product hydrolysates obtained with papain and Protamex^TM^, with respective values of 256.63 mg/g and 252.25 mg/g. The smallest amount of free amino groups was for the samples hydrolyzed by pepsin, ME (134.63 mg/g), and SE (127.72 mg/g).

Regarding DH and the average PCL, samples showed differences between fish species and the enzyme used for preparing hydrolysates. The DH obtained by the different samples ranged from 34.45% to 55.61%. Higher DH values were found for the sardine by-products hydrolyzed with papain and Protamex^TM^, which had DH percentages of 55.39% and 55.61%, respectively. The lowest DH values were for the samples hydrolyzed by pepsin, ME (34.45%), and SE (34.74%). Regarding the DH, the Papain and Protamex™ hydrolysates obtained similar PCL values but were higher for the mackerel than for the sardine samples. Specifically, the mackerel hydrolysates showed PCL values of 2.14 and 2.24 for papain and Protamex™, respectively, while these values dropped to approximately 1.80 for the sardine samples. Pepsin hydrolysates exhibited the highest PCL values, with no differences between raw materials. In both cases, the obtained PCLs were 2.90.

### 2.2. Antioxidant Activity

**Figure 1:**
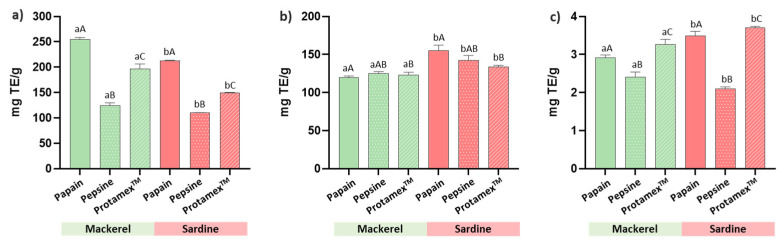
Antioxidant activity of the Atlantic mackerel and sardine by-product hydrolysates samples (100 mg/mL) as measured by (**a**) DPPH radical scavenging activity, (**b**) ABTS radical scavenging capacity, and (**c**) FRAP activity. Bars represent the means and their SD (*n* = 3). Different lower-case letters indicate the significant differences between raw materials, and different upper-case letters denote significant differences among enzymes (*p* < 0.05).

The DPPH radical scavenging activity of hydrolysates is shown in Figure 1a. According to this method, significant differences were observed depending on the raw material and the enzyme used to produce hydrolysates. The hydrolysates prepared from the mackerel by-products showed greater DPPH scavenging activity than the sardine hydrolysates. Considering the enzyme, the greatest DPPH radical scavenging activity was observed for papain, with values of 254.60 and 212.82 mg TE/g for the Atlantic mackerel and sardine by-products, respectively. These were followed by the Protamex™ hydrolysates prepared from Atlantic mackerel (124.65 mgTE/g) and sardine (149.87 mgTE/g) waste. The lowest DPPH response was for the pepsin hydrolysates, with values of 124.65 mgTE/g and 109.99 mgTE/g when the starting material was Atlantic mackerel and the sardine by-product, respectively.

Regarding ABTS radical scavenging activity, the sardine by-product hydrolysates displayed higher reducing power than the mackerel samples when using the three enzymes (Figure 1b). The higher antioxidant capacity achieved by this method was observed for the sardine samples produced by papain (155.44 mgTE/g), followed by the sardine hydrolysates obtained with pepsin (142.20 mgTE/g) and, finally, by the sardine samples produced with Protamex™ (134.11 mgTE/g). Instead, small differences were observed in the Atlantic mackerel hydrolysates prepared with pepsin, Protamex™, and papain, which exhibited ABTS scavenging activity of 124.98 mgTE/g, 123.38 mgTE/g, and 120.07 mgTE/g, respectively.

The FRAP activity of hydrolysates is shown in Figure 1c. According to this method, the samples’ antioxidant capacity is determined by their ability to reduce iron (III) to iron (II). As in the DPPH assay, significant differences in FRAP antioxidant activity were observed depending on the raw material and the enzyme used to produce hydrolysates (Figure 1). However, unlike the DPPH results, the sardine hydrolysates displayed higher reducing power than the Atlantic mackerel hydrolysates. The highest antioxidant capacity, as measured by the FRAP assay, was for the sardine hydrolysates obtained with Protamex™ and papain, which displayed values of 3.70 mgTE/g and 3.49 mgTE/g, respectively. These were followed by the mackerel hydrolysates obtained with Protamex™ and papain, with values of 3.27 mgTE/g and 2.92 mgTE/g, respectively. Last, the samples hydrolyzed with pepsin had the lowest ABTS-reducing power, with values of 2.41 mgTE/g for the sardine hydrolysate and 2.10 mgTE/g for the mackerel sample.

### 2.3. Potential Antidiabetic Activity

**Figure 2:**
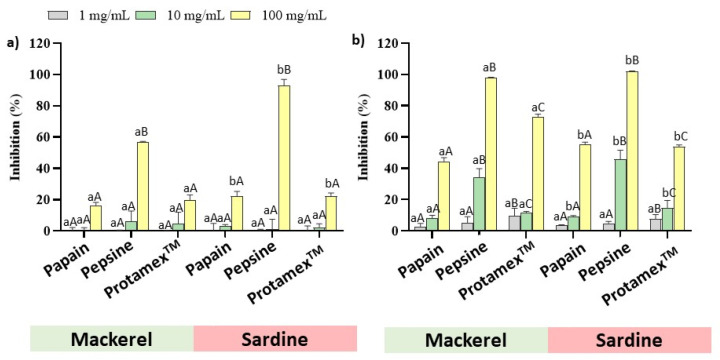
Inhibitory effects of Atlantic mackerel and sardine by-product hydrolysates against α-amylase (**a**) and α-glucosidase (**b**) activity. Bars represent the means of three replicates and their standard deviation (SD). Different lower-case letters indicate significant differences between raw materials, and different upper-case letters denote significant differences among enzymes (*p* < 0.05).

The α-amylase and α-glucosidase inhibitory effects of fish by-products are shown in Figure 2. All the samples inhibited α-amylase activity at the highest tested concentration (100 mg/mL). A stronger effect was found for SE and ME, which obtained an inhibition percentage of 93% and 57%, respectively. Instead, no differences were observed between the Papain and Protamex^TM^ hydrolyzed samples, and their inhibition values remained within the range, between 16% and 22%. Moreover, all the samples showed α-glucosidase activity at the tested concentrations. As occurred in the α-amylase activity assay, similar values were obtained depending on the enzyme used during the hydrolysis process. The most marked effects were observed for the pepsin-hydrolyzed samples. The α-glucosidase inhibition values at the 10 mg/mL concentration were 46% and 35% for ME and SE, respectively, and these values increased to percentages close to 100% for the same samples at the 100 mg/mL concentration. The inhibition percentages of the Protamex^TM^ hydrolysates at the 10 mg/mL concentration were 12% and 15% for the mackerel and sardine samples, respectively, and 73% and 54% for the 100 mg/mL concentration. Finally, values ranged between 8% and 9% for the papain-hydrolyzed samples at the 10 mg/mL concentration, and between 35% and 46% at the 100 mg/mL concentration, respectively.

### 2.4. Multivariate Analysis

A multifactor analysis of variance (ANOVA) was performed to analyze the influence of fish species, enzyme type, and their interaction on the degree of proteolysis and the bioactive properties of hydrolysates. No α-amylase activity was observed at low hydrolysate concentrations (1 and 10 mg/mL); thus, the statistical analysis was not applied in these cases. As shown in Table 2, both factors and their interaction significantly affected all the analyzed parameters, except for DPPH scavenging activity, which was not influenced by the type of enzyme used during hydrolysis. The strongest effect of the fish species factor appeared for α-amylase inhibitory activity, FRAP function, and free amino groups. The most pronounced effect of the enzyme factor was on α-amylase and α-glucosidase inhibitory activities and FRAP antioxidant action. The interaction between both factors affected mainly α-glucosidase inhibition, total protein content, and α-amylase inhibitory activity, and in that order.

**Figure 3:**
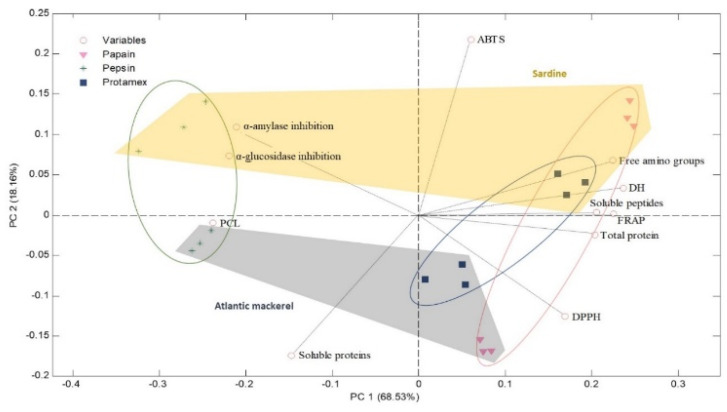
PCA biplot of the physicochemical and bioactive properties of hydrolysates. The drawn ellipses and color stains highlight the natural samples clustering for enzyme type and fish species, respectively, but do not represent statistical significance. Abbreviations: PCL, Peptide Chain Length; ABTS, ABTS radical scavenging activity; DH, degree of hydrolysis; FRAP, Ferric-Reducing Antioxidant Power; DPPH, DPPH radical scavenging capacity.

A PCA was performed to visualize the sample distribution and the relation among variables (Figure 3). The results showed that 85.8% of the variance in the dataset was described by two principal components, which allowed differentiation of the proteolytic enzymes, and the raw material used to produce hydrolysates. Principal component 1 (PC1) explained 65.84% and accounted for the variability produced by the factor enzyme type. The variables contributing the most to PC1 were free amino groups, DH, soluble peptides, total protein, and, to a lesser extent, DPPH activity with a positive weight, and α-amylase and α-glucosidase activities with a negative weight. As observed in the PCA score plot, pepsin hydrolysates were grouped on the left of the graph together with the variables α-amylase and α-glucosidase activity and soluble proteins and PCL. The Papain and Protamex^TM^ samples were clustered on the right of the graph with free amino groups, DH, soluble peptides, and total protein, and the three analyzed antioxidant parameters (ABTS, FRAP, and DPPH scavenging activities). PC2 was responsible for 19.96% and contained the variance produced by the fish species by-product type. The most important variables for PC2 were ABTS with a positive weight, and soluble proteins and DPPH assay with a negative weight. The sardine samples were clustered in the upper part of the PCA score plot, together with α-amylase and α-glucosidase activities, ABTS scavenging activity, free amino groups, DH, soluble peptides, and FRAP function. On the contrary, the mackerel hydrolysates were grouped in the lower part of the plot, along with the variable TCA-soluble proteins and DPPH scavenging activity.

**Figure 4:**
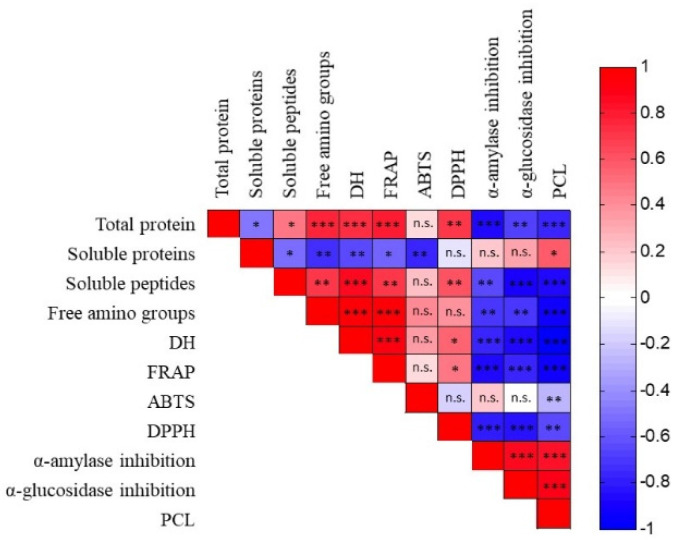
Pearson correlation coefficient r and p values among the variables analyzed in the study. Abbreviations: PCL, Peptide Chain Length; ABTS, ABTS radical scavenging activity; DH, degree of hydrolysis; FRAP, Ferric-Reducing Antioxidant Power; DPPH, DPPH radical scavenging capacity. ns: not significant, * *p* < 0.05, ** *p* < 0.01, *** *p* < 0.001.

Pearson’s correlation analysis was performed to determine the strength of the linear relation among the different variables. As shown in the Pearson correlation matrix (Figure 4), a significant linear relation was found between a major part of the analyzed variables. The strongest positive correlations were noted between soluble peptides and DH (r = 0.8598; *p* = 0.0000), free amino groups and DH (r = 0.9582; *p* = 0.0000), free amino groups and FRAP (r = 0.9425; *p* = 0.0000), DH and FRAP (r = 0.9229; *p* = 0.0000), PCL and α-amylase inhibition activity (r = 0.8247; *p* = 0.0000), and PCL and α-glucosidase inhibition activity (r = 0.8811; *p* = 0.0000) α-amylase and α-glucosidase inhibition activities (r = 0.8713; *p* = 0.0000). A strong negative correlation was found between total protein and α-amylase inhibition activity (r = −0.8480; *p* = 0.0000), soluble peptides and PCL (r =; *p* = 0.0000), soluble peptides and PCL (r = −0.8477; *p* = 0.0000), soluble peptides and α-glucosidase inhibition activity (r = −0.9039; *p* = 0.0000), free amino groups and PCL (r = −0.9291; *p* = 0.0000), DH and PCL (r = −0.9879; *p* = 0.0000), DH and α-glucosidase inhibition activity (r = −0.8543; *p* = 0.0000), PCL and FRAP (r = −0.9190; *p* = 0.0000), FRAP and α-amylase inhibition activity (r = −0.8487; *p* = 0.0000), and DPPH and α-glucosidase inhibition activity (r = −0.8251; *p* = 0.0000).

## 3. Discussion

Protein hydrolysates are industrially produced by chemical, bacterial fermentation, autolytic, or enzymatic hydrolysis methods [21,22]. However, enzymatic hydrolysis is the most widely used procedure for food and biomedical applications because it uses mild and controlled conditions, obtaining more efficient and reproducible results [23]. Enzymatic hydrolysis results in amino acids and peptides of varied sizes depending on the targeting of specific peptide cleavage bonds of the employed enzyme [24]. Most of the enzymes used for this purpose originate from plants, animals, or microbial sources [25]. The enzyme origin determines the specificity degree of proteolytic enzymes by playing a crucial role in the hydrolysis process and the properties of the resulting hydrolysate [22]. In this study, we produced Atlantic mackerel and sardine by-product hydrolysates using enzymatic hydrolysis. The fish by-product hydrolysates obtained in our study exhibited a high protein content, similar to that reported by other authors when analyzing protein hydrolysates produced from sardine and mackerel by-products or discards [13,26,27].

Hydrolysis was performed by three proteolytic enzymes: papain, pepsin, and Protamex^TM^. Papain is a cysteine protease extracted from the raw fruit of the papaya plant. Protamex^TM^ is an endopeptidase produced from *Bacillus subtilis* fermentation. Pepsin is an aspartate protease, active under acidic conditions, and is the main protease from human gastric juice [28,29]. Plant-origin enzymes have been described to exhibit broader specific action than animal-origin enzymes, while a higher diversity appears for microbial proteases [25]. Independent of the type of raw material employed, small differences were observed between the papain and Protamex^TM^ hydrolysates in our study, while bigger differences were found for the pepsin hydrolysates. The Papain and Protamex^TM^ samples exhibited a higher DH than the pepsin hydrolysates, and consequently lower PCL values and soluble protein content, but higher TCA-soluble peptides and free amino groups content. Instead, the pepsin hydrolysates had a lower DH and, thus, a higher content in soluble proteins and lower content in TCA-soluble peptides and free amino groups, as well as lower PCL values. These results agree with the specificity degree described for each enzyme [30]. Papain shows specificity by cleaving peptide bonds from hydrophobic regions, such as amino acids alanine, leucine, phenylalanine, or tyrosine [31]. Similarly, Protamex^TM^ shows broad specificity for hydrophobic amino acids [29]. Instead, pepsin displays a higher specificity degree and only cleaves proteins at the phenylalanine and leucine bond [25]. Accordingly, we observed that the DH decreased, and the produced peptides were larger when the enzyme specificity increased.

Protein hydrolysates have many applications, including their use as nutritional supplements, flavor enhancers, growth media, beverages, cosmetics, and pharmaceutical ingredients, among others [32,33,34]. More recently, the use of protein hydrolysates as a source of functional and bioactive peptides has been proposed [33,35,36]. Oxidation reactions are one of the main sources of food deterioration during manufacturing, storage, distribution, and final preparation [37]. These reactions affect the quality of food products because they may produce nutrients, texture, water-holding capacity, color and flavor impairment, and toxic substances may form [38]. An imbalance between reactive oxygen species (ROS) production and antioxidant defenses in the organism can lead to cumulative damage in different cell components, including proteins, lipids, and nucleic acids, which results in oxidative stress [39]. Oxidative stress has been related to aging and a variety of chronic diseases, such as cancer, and cardiovascular and neurodegenerative diseases, among others [40,41]. Antioxidant molecules play a crucial role in reducing oxidative processes in both food systems and pharmaceutical products and preventing the harmful effects of ROS in the human body, thus reducing the risk of oxidative stress-mediated chronic diseases [42 However, the potential toxicity of synthetic antioxidants and their undesirable effects have been recently considered [42]. In this context, the use of natural resources to obtain antioxidants capable of extending the shelf life of foods and reducing the risk of chronic diseases is interesting from a technological, economic, and health point of view [37]. However, these antioxidants could interact with other compounds present in foods and biological systems, exhibiting synergistic or antagonistic effects. This should be especially considered when incorporating them into foods, since antioxidant peptides may also lose their activity due to the interaction with other components of the food matrix, such as carbohydrates, lipids, trace metals, or other antioxidants, or during food processing operations.

More than one antioxidant assay must be performed to gain an in-depth understanding of the antioxidant properties of antioxidant compounds and their mechanism of action [43]. Accordingly, three commonly applied antioxidant methods were used in this study to evaluate the antioxidant properties of the fish by-product hydrolysates: the DPPH and ABTS assays, which measure free radical scavenging capacity; and the FRAP assay, which quantifies ferric reducing capacity. Our results showed that antioxidant activity was affected by the raw material and the enzyme used to produce hydrolysates. According to the DPPH method, the mackerel by-products produced more antioxidant hydrolysates than the sardine waste. García-Moreno et al. (2014) [26] evaluated different discarded species, including sardine and horse mackerel, to obtain fish protein hydrolysates. The highest DPPH scavenging activity was observed in sardine hydrolysates, followed by mackerel. These results also agree with those reported by Morales-Medina et al. (2016) [44] who hydrolyzed sardine and horse mackerel protein using Alcalase or trypsin. However, environmental and biological factors can substantially influence the composition of fish by-products, thereby determining the bioactive properties of the hydrolysates and their potential applications.

The Papain samples displayed the highest antioxidant capacity, followed by Protamex™, and finally pepsin. Similarly, by following the FRAP method, pepsin produced the lowest antioxidant hydrolysates, while the effect of raw material on the antioxidant properties of hydrolysates depended on the employed enzyme type. As previously mentioned, the Papain and Protamex™ samples exhibited a higher DH than the pepsin hydrolysates and consequently had lower PCL values. Indeed, as shown by the multivariate analysis, hydrolysates’ antioxidant capacity correlated with a high content of free amino groups, a high DH, and low PCL values. Based on these results, protein hydrolysates containing mainly low-molecular-weight peptides exhibit the highest antioxidant capacity. Indeed, peptides with low molecular weight have been reported to be the main contributors to antioxidant activity in several fish hydrolysates due to their hydrophilic nature and higher exposure to antioxidant amino acid residues [33,45,46]. At the same time, the lower scavenging capacity of high-molecular-weight peptides has been attributed to the possible steric hindrance caused by their size, which leads to increased peptide repulsion [47]. However, some authors have also observed the low functional and antioxidant properties of fish protein hydrolysates for high DH values [19,46]. This discrepancy among studies may be a consequence that, together with low molecular weight, other characteristics of protein hydrolysates and peptide fractions, including the presence of amino acids like Leu or Val in their N-terminal regions, nucleophilic sulfur-containing amino acid residues (Cys and Met), hydrophobic amino acids, aromatic amino acids, and the imidazole ring-containing His, have also been related to significant antioxidant properties [46,48,49]. Yet the exact relation between the structure of peptides and their antioxidant activity is not fully understood [49].

The different results were obtained depending on the assay type. Unlike the DPPH and FRAP methods, according to the ABTS assay, the sardine hydrolysates performed higher antioxidant capacity than the mackerel samples, and no differences were found between the pepsin and Protamex™ hydrolysates. The method used to measure and calculate antioxidant activity can significantly impact the results due to the complexity of oxidation reactions [50]. Other authors have also found discrepancies when evaluating the antioxidant activity of samples by distinct methods [51,52]. Different mechanisms have been proposed for the antioxidant activity of fish-origin proteins and peptides, including the free radicals scavenging activity of specific amino acids/terminals (aromatic and H, P, M, L, Y, and C), sequestration and chelation of ROS, or inhibition of lipid and protein oxidation [53]. The differences observed in samples’ antioxidant activity depending on the employed assay can be attributed to each method’s mechanisms of action and sensitivities [54]. The DPPH method focuses on electron-donating capacity in a nonpolar medium, while the ABTS is more sensitive to certain antioxidant compounds in a hydrophilic medium. The FRAP assay evaluates the reducing capacity of antioxidants by focusing on the ferric to ferrous ions. Moreover, these assays differ depending on the oxidant and target/probe species, reaction conditions, standard compounds, and the form in which results are expressed [53]. All this reveals the importance of selecting the appropriate assay to evaluate the antioxidant activity of peptides depending on their chemical properties and intended application.

Diabetes is a chronic metabolic disease characterized by high blood glucose levels that can lead to serious complications over time, including damage to the heart, kidneys, eyes, nerves, and the peripheral vascular system [55]. The common type of diabetes is type 2 diabetes, which appears when the body does not produce enough insulin or becomes resistant to it [56]. The incidence of type 2 diabetes has remarkably increased in the last few decades because of obesity, a sedentary lifestyle, and population aging [55]. According to the IDF Diabetes Atlas, 537 million adults had diabetes in 2021, which accounted for 10.5% of the adult population [57]. This number is expected to increase to 643 million by 2030 and 783 million by 2045 [57]. Furthermore, this disease is responsible for substantial care costs and many deaths every year, with 6.7 million in 2021 [57].

Carbohydrate-hydrolyzing enzymes α-amylase and α-glucosidase are key in blood glucose regulation levels because they control carbohydrate digestion and absorption [58]. The α-amylase enzyme starts the carbohydrate digestion process by the hydrolysis of 1,4-glycosidic linkages of polysaccharides, such as starch or glycogen, to oligosaccharides and disaccharides [59]. The α-glucosidase enzyme, located in the surface membrane of small intestinal cells, catalyzes the hydrolysis of α-1,2, α-1,4, and α -α-1,6-glucosidic bonds in oligosaccharides and disaccharides, which results in the release of absorbable monosaccharides that enter the bloodstream [58]. So, the inhibition of these enzymes is used to lower blood glucose levels and the risk of developing diabetes. Nowadays, only three inhibitors of carbohydrate-hydrolyzing enzymes are approved to treat diabetes: acarbose, miglitol, and voglibose [60]. However, these drugs cause undesirable gastrointestinal side effects, such as vomiting, flatulence, urinary tract infection, and liver disorders, among others [61]. Consequently, searching for natural compounds that exhibit inhibition activity on these enzymes while presenting fewer adverse effects is extremely interesting.

The protein hydrolysates obtained from the flesh and by-products of different fish species, such as salmon, blue whiting, Sind sardine, or Mueller’s pearlside, have been previously utilized to produce potent antihyperglycaemic agents [51,62,63,64,65]. Yet, there is limited information about the production of α-amylase and α-glucosidase inhibitory peptides through enzymatic hydrolysis. So further research is necessary to isolate active sequences produced by proteases with varying specificities [66].

In this study, the mackerel and sardine by-product hydrolysates displayed in vitro antidiabetic effects by inhibiting both α-amylase and α-glucosidase in a concentration-dependent manner. Interestingly, these effects were determined by the raw material and the enzyme used during the hydrolysis process. The α-glucosidase inhibitory effects of the fish by-product hydrolysates obtained in this work were stronger than those reported in other studies with similar fish species. As previously reported, 10 mg/mL of the hydrolysates produced in this work exhibited a range of inhibition percentages between 9.1% and 45.8%, and between 8.3% and 34.5% for sardine and mackerel, respectively. Instead, Amini Sarteshnizi et al. (2021) [65] found that 20 mg/mL of Sind sardine hydrolysates displayed 4.69% α-glucosidase inhibitory activity for pepsin hydrolysates, while no inhibition was observed in the papain-hydrolyzed samples. In the hydrolysates produced from whole Atlantic horse mackerel using Alcalase, Henriques et al. (2021) [51] obtained an inhibitory activity of 50% for 143.3 mg/mL against α-glucosidase. Instead, at the 100 mg/mL concentration of the Atlantic mackerel by-products hydrolysates, we found that the α-glucosidase inhibition percentages ranged from 46% to 102%. These results highlight the interest in utilizing fish by-products to obtain hydrolysates and peptides with bioactive properties.

When considering both α-amylase and α-glucosidase inhibitory activity, the maximum inhibition percentages were for the pepsin-hydrolyzed samples, while small differences were observed between the papain and Protamex™ samples. Enzymes produce peptides of varying molecular sizes, hydrophobicity, and amino acid sequences based on their cleavage specificity, and these properties have been demonstrated to determine their antioxidant and in vitro antidiabetic effects [48,65]. In this study, we found a negative correlation between α-amylase and α-glucosidase activities and the parameters of free amino groups, DH, soluble peptides, and total protein. However, a positive correlation was observed between these activities and PCL. Together, these results suggest that high-molecular-weight peptides more efficiently inhibit α-amylase and α-glucosidase enzymes.

As previously mentioned, pepsin exhibits a higher specificity degree in the amino acid sequences, producing protein hydrolysates with lower DH and PCL values than papain and Protamex™. Unlike the results found in our work, other authors have reported a positive relation between DH and antidiabetic activity. Li-Chan et al. (2012) [67] studied the antidiabetic properties of peptides derived from Atlantic salmon skin gelation hydrolyzed by alcalase (a serine protease), bromelain (a cysteine protease), and Flavourzyme (an exo- and endopeptidase complex) by measuring dipeptidyl-peptidase IV (DPP-IV)-inhibitory activity. According to their results, the flavorzyme hydrolysate with a 6% enzyme/substrate ratio showed the highest DH and the greatest DPP-IV-inhibitory activity [67]. Similarly, Amini Sarteshnizi et al. (2021) [65] observed a higher inhibitory response against α-glucosidase in Sind sardine hydrolysates when produced with pepsin than papain. Instead, these authors found significantly lower α-amylase inhibition for the pepsin than the papain hydrolysate [65]. However, other studies suggest that amino acid sequence rather than the molecular weight correlates to bioactivity [68]. In particular, structural properties from the peptides present in hydrolysates, such as hydrophobic residues number, ratio of hydrophobic residues from total, high concentrations of Glu, Ala, Lys, Arg, and His residues, high amino acid content or presence of L and P in the last two positions at the Nt, have been proposed as important features for antidiabetic activity [66,69,70].

The mechanism by which peptides inhibit α-amylase and α-glucosidase activity is not fully understood. However, it has been suggested that non-sugar compounds, such as polypeptides, can bind to the enzyme’s active site through hydrophobic interactions due to the presence of hydrophobic amino acids at the N-terminal of peptides, which allows them to exert their inhibitory activity [35,48]. Hence, higher hydrophobic amino acid content has been found for pepsin than for other protease hydrolysates like alcalase and papain [65]. Additionally, larger peptides have been observed to have more hydrophobic binding sites than smaller peptides [65]. All these findings support our results.

## 4. Materials and Methods

### 4.1. Chemicals

Pepsin of porcine origin, Coomassie Brilliant Blue G-250, bovine serum albumin (BSA), L-tyrosine, L-leucine, 2,4,6-trinitrobenzene sulfonic acid (TNBS), potassium persulfate, sodium acetate trihydrate, 2,4,6-tri(2-pyridyl)-s-triazine (TPTZ), α-amylase from porcine pancreas (A3176-500KU), α-glucosidase from Saccharomyces cerevisiae (G5003-1KU), potato starch, 3,5-dinitrosalicylic acid, acarbose, phenol solution and p-nitrophenyl α-D-glucopyranoside (p-NPG) were acquired from Sigma-Aldrich Co. (Burlington, MA, USA). Papain was purchased from Biocon (Barcelona, Spain), and Protamex^TM^ (Tepic, Mexico) was supplied by Novozymes (Bagsværd, Denmark). Trichloroacetic acid (TCA), sodium potassium tartrate tetrahydrate, and glacial acetic acid came from LabKem (Barcelona, Spain). 2,2’-azino-bis(3-ethylbenzothiazoline-6-sulfonic acid) diammonium salt (ABTS) was purchased from Applichem (Hessen, Germany). Ethanol, methanol, ascorbic acid, ferric chloride (FeCl_3_), HCl, NaOH, and sodium organic salts were supplied by Scharlab (Barcelona, Spain). 2,2-diphenyl-1-picrylhydrazyl (DPPH) was acquired from TCI Chemicals (Tokyo, Japan). Trolox was obtained from Acros Organics (Geel, Bélgica). All the chemicals used in the experiments were of analytical grade.

### 4.2. Raw Material

Fresh samples of Atlantic mackerel and sardine were acquired from a local market (Valencia, Spain). Upon arrival at the laboratory, fish were eviscerated, filleted, and the muscle was manually separated to obtain the fish by-products. Mackerel by-products consisted of heads, bones, thorns, skin, fins, and viscera. In the case of sardine, the skin was not separated from the muscle, and by-products consisted of heads, bones, thorns, fins, and viscera. Fish handling resulted in 43% and 41% of Atlantic mackerel and sardine by-products.

By-products from each fish species were collected, weighed, washed with distilled water, homogenized, and frozen at −40 °C in sealed plastic bags (250 g). The proximate composition of fish by-products was determined in homogenized fish by-products before freezing according to the AOAC Methods 950.46, 991.36, 928.08, and 920.153, respectively [71]. The proximate composition analysis of samples resulted in 76% moisture, 16% protein, 3% fat, and 4% ash for the mackerel by-products, and 72% moisture, 15% protein, 6% fat, and 5% ash for the sardine by-product samples.

### 4.3. Enzymatic Hydrolysis

**Figure 5:**
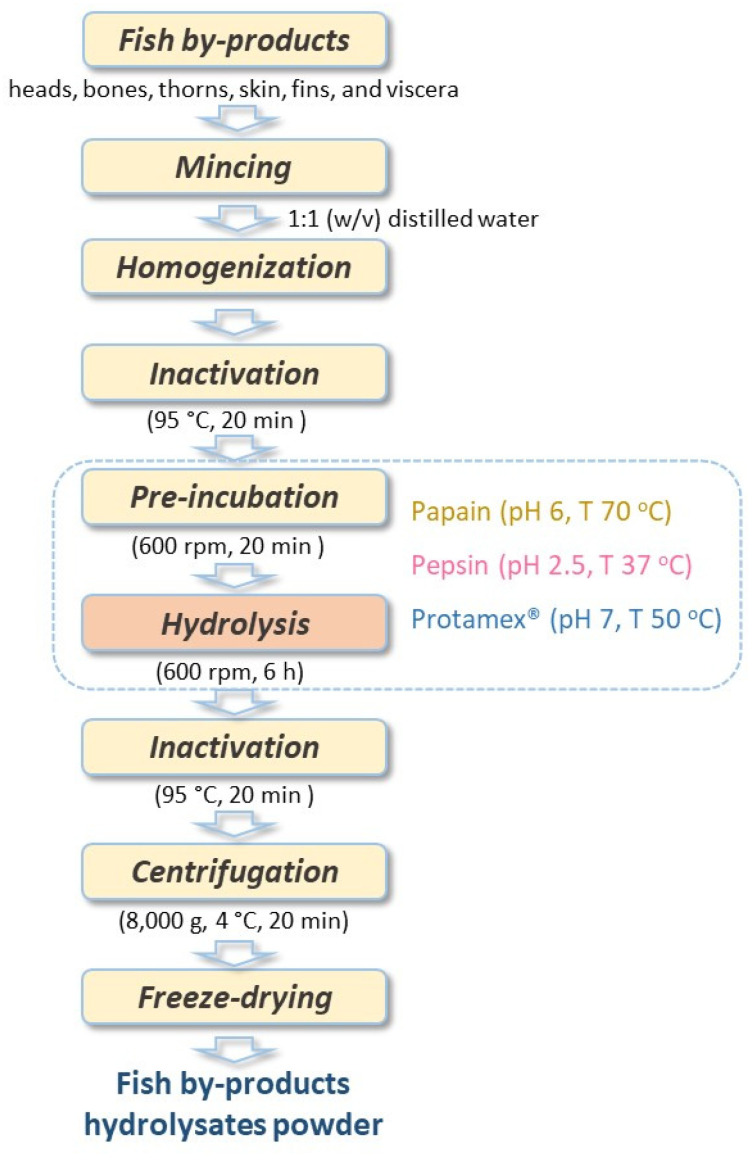
Schematic representation of the enzymatic hydrolysis process.

The schematic hydrolysis process used in this study is presented in Figure 5. For each hydrolysis reaction, 250 g of fish by-products, previously thawed overnight in a refrigerator at 4 °C, was mixed 1:1 (*w*/*v*) with distilled water, and then homogenized in a Thermomix food processor (Thermomix, Wuppertal, Germany). The mixture was heated at a temperature of 95 °C for 20 min in a boiling water bath to inactivate any native enzymes present in the raw material. Before hydrolysis, solutions were preheated to different reaction temperatures and adjusted to distinct pHs depending on the employed enzyme. Three different commercial proteases were used for the enzymatic hydrolysis in this study: papain (pH 6, T 70 °C) of plant origin, pepsin of animal (pH 2.5, T 37 °C) origin, and Protamex^TM^ (pH 7, T 50 °C) of bacterial origin. After the 20 min pre-incubation, the reaction was initiated by adding papain, pepsin, or Protamex™ to the by-product mixture (1.5%, *w*/*w*). Then, samples were hydrolyzed with stirring (600 rpm) for 6 h, the temperature was checked, and the pH was maintained by adding 1 N NaOH. At the end of the hydrolysis process, the temperature was increased to 95 °C for 20 min to deactivate enzymes. The hydrolysate solutions were cooled to room temperature and centrifuged at 8000× *g* at 4 °C for 20 min. Finally, supernatants were paper-filtered to remove impurities, frozen at −40 °C overnight, and freeze-dried (LyoQuest-55, Telstar, Barcelona, Spain) for 48 h. The freeze-dried powders were stored at −40 °C for further analysis.

### 4.4. Proteolysis Analysis

The Kjeldahl method was used to determine the total protein content according to AOAC 928.08 [72]. Total soluble proteins, TCA-soluble peptides, free amino groups and degree of hydrolysis were analyzed following the protocols described by Gallego et al. (2020) [73].

Total soluble protein quantification was performed based on Bradford’s assay method. Briefly, 0.1 g of the hydrolysate sample was diluted in 1 mL of distilled water. Then, 40 µL of sample solution and 2 mL of the Bradford reagent were mixed and incubated at room temperature for 5 min. Absorbance was read at 595 nm by a UV–visible spectrophotometer (Helios Zeta, Thermo Scientific, Hemel Hempstead, UK) using BSA as a standard. The results were expressed as mg BSA/g sample.

For the TCA-soluble peptide content determination, 50 µL of the sample was mixed with 450 µL of TCA (5% *w*/*v*) and incubated at 4 °C for 60 min. After centrifugation (8000× *g*, 10 min), the absorbance of the supernatant was measured at 280 nm. The results were expressed as mg L-tyrosine equivalents/g sample.

The free amino group content was quantified using the TNBS method. For this purpose, 40 µL of sample and 320 µL of sodium phosphate buffer (0.2 M, pH 8.2) were mixed with 320 µL of TNBS (0.1%, *v*/*v*). The mixture was incubated at 50 °C for 60 min. Then, 640 µL of HC1 (0.1 N) was added. After a 30 min incubation, absorbance was read at 340 nm. The results were expressed as mg L-leucine equivalents/g sample.

The degree of hydrolysis (DH) was determined based on the amount of free amino groups conforming to the TNBS method. DH was calculated using the formula:

DH = (H_t_ − H_0_)/(H_c_ − H_0_),

where H_t_ is the free amino groups’ concentration in the hydrolysate samples, H0 is the free amino groups’ concentration before hydrolysis, and H_c_ is the free amino group content after completing the hydrolysis of the samples. The total hydrolysis of samples was performed by dissolving 100 mg of the hydrolysate in 2.5 mL of 6 N HCl at 110 °C for 24 h.

The average Peptide Chain Length (PCL) was estimated from the DH (%) values as described by Adler-Nissen and Olsen (1979) [74], according to the following equation:

PCL = 100/DH (%).

### 4.5. Antioxidant Activity

#### 4.5.1. DPPH Radical Scavenging Capacity

The scavenging capacity of hydrolysates against the DPPH radical was determined based on the method developed by Blois (1958) [75]. A volume of 6 µL of the sample solution (0.1 g/mL) was mixed with 294 µL of methanol and 2.7 mL of DPPH solution (0.003%, *w*/*v*) and incubated for 60 min in the dark. Absorbance was measured at 517 nm. To ensure the reliability and validity of the assay, positive controls (ascorbic acid 0.5 mM), negative controls (methanol and DPPH solution), and blanks (methanol) were included. The results were expressed as mg Trolox equivalents (TE)/g sample.

#### 4.5.2. ABTS Radical Scavenging Activity

The ABTS radical scavenging activity was assessed according to the protocol described by Gallego et al. (2020) [76]. ABTS+ radical was produced by reacting ABTS (7 mM) with potassium persulfate (2.45 mM) overnight. Before the experiments, the ABTS^+^ solution was diluted with phosphate-buffered saline (PBS 50 mM, pH 7.4) to adjust absorbance to 0.7 (±0.05) at 734 nm. Then, 10 µL of sample solution (0.1 g/mL) was mixed with 990 µL of ABTS+ solution and allowed to stand at room temperature for 6 min. A positive control (ascorbic acid 1 mM in PBS), a negative control (the mixture without antioxidant), and a blank (the solvent without ABTS) were included to ensure the reliability and accuracy of the results. Absorbance was measured at 734 nm, and the results were expressed as mg Trolox equivalents (TE)/g sample.

#### 4.5.3. Ferric-Reducing Antioxidant Power (FRAP)

FRAP activity was evaluated using the modified method developed by Benzie and Strain (1996) [77]. The FRAP reagent was first prepared by mixing acetate buffer (0.3 M, pH 3.6), TPTZ (10 mM), and FeCl_3_·6H_2_O (20 mM) at the ratio of 10:1:1. Then, 30 µL of sample solution (0.1 g/mL) were mixed with 30 µL of acetate buffer (0.3 M, pH 3.6) and 900 µL of the FRAP reagent. The mixture was incubated at 37 °C for 30 min in the dark. Then, the absorbance of the samples was measured at 595 nm. The results were expressed as mg Trolox equivalents (TE)/g sample.

### 4.6. Potential Antidiabetic Activity

#### 4.6.1. α-Amylase Inhibition Assay

The α-amylase inhibition assay was performed following the protocol described by Arnal et al. (2023) [78]. According to this method, 50 µL of sample solution (1, 10, and 100 mg/mL) and 50 µL of α-amylase 13 U/mL in 0.02 M sodium phosphate buffer (6 mM NaCl, pH 6.9) were incubated at room temperature for 10 min. Next, 50 µL of 1% starch solution in sodium phosphate buffer was added, and the mixture was incubated for 10 min at room temperature. Then, 100 µL of dinitrosalicylic acid reagent (1% 3,5-dinitrosalicylic acid, 0.2% phenol, 0.05% Na_2_SO_3_, and 1% NaOH in aqueous solution) were added, and samples were heated to 95 °C for 5 min. After cooling, 1 mL of deionized water was added, and absorbance (A) was measured in a spectrophotometer at 570 nm. A sample blank was prepared by replacing the sample with sodium phosphate buffer. Acarbose (1 mM) was used as a positive control. The results were expressed as a percentage of inhibition according to the formula:

Inhibition (%) = ((A_control_ − (A_sample_ − A_sample blank_)/A_control_)) × 100.

#### 4.6.2. α-Glucosidase Inhibition Assay

The assessment of α-glucosidase inhibitory activity was assessed according to Arnal et al. (2023) [78]. For this assay, 400 µL of sample and 200 µL of α-glucosidase 0.5 U/mL in 0.1 M sodium phosphate buffer (pH 6.8) were incubated at 37 °C for 5 min. Then, 200 µL of 1.25 mM p-NPG solution in 0.1 M sodium phosphate buffer was added, and the mixture was incubated at 37 °C for 20 min. Absorbance was read at 405 nm. Acarbose (4 mg/mL) was used as a positive control. The results were expressed as a percentage of inhibition using the formula described for the α-amylase inhibition assay.

### 4.7. Statistical Analysis

All the analyses were performed in triplicate. A multifactor analysis of variance (ANOVA) was carried out to test the effect of raw material and the enzyme on proteolysis and the bioactive properties of hydrolysates using Statgraphics Centurion XVIII (Statpoint Technologies, Inc., Warrenton, VA, USA). The Tukey HSD (Tukey’s Honest Significant Difference) procedure was used to test for differences between means at the 5% significance level. A multivariate principal component analysis (PCA) was performed to explore the effect of the different variables on the collected data using the MATLAB^®^ PLS Tool-box, 6.3 (Eigenvector Research Inc., Manson, WA, USA).

## 5. Conclusions

This study has demonstrated the potential of Atlantic mackerel and sardine waste as a source of bioactive peptides, highlighting the opportunity for revalorizing these by-products. Our results show that the characteristics of protein hydrolysates heavily depend on the enzyme used and the fish species. Thus, depending on the required functionality, different conditions must be employed. Specifically, sardine by-products and pepsin would be preferable for producing hydrolysates with antidiabetic potential, and mackerel by-products and enzymes papain and Protamex^TM^ could be used for producing peptides with antioxidant capacity. Accordingly, Atlantic mackerel and sardine by-products may be used as a natural source of bioactive peptides to prepare functional food ingredients, nutraceuticals, or pharmaceutical agents. Further research is required to identify and characterize the peptides responsible for the bioactivity of hydrolysates, to evaluate the interaction of these compounds within the food matrix, and to demonstrate their properties in vivo. Employing fish waste as a natural source of bioactive hydrolysates and peptides for different applications is an opportunity for its revalorization, which provides the sector with economic benefits and solves pollution problems that derive from fish processing operations. However, the industrial viability of these initiatives should be addressed through comprehensive and easily scalable models that encourage industries to invest more in the valorization of by-products, thus contributing to a net-zero emissions economy.

## Figures and Tables

**Table 1:** The total protein content (TP), total soluble proteins (TSP), TCA-soluble peptides (TCA-SP), free amino groups (FAG), degree of hydrolysis (DH), and peptide chain length (PCL) of fish by-product hydrolysates.

Raw Material	Enzyme	TP (%)	TSP (mg/g)	TCA-SP (mg/g)	FAG (mg/g)	DH (%)	PCL
	Papain Pepsine Protamex^TM^	52.55 (0.83) ^aA^	3.13 (0.11) ^aA^	141.92 (3.60) ^aA^	166.57 (3.56) ^aA^	46.65 (1.00) ^aA^	2.14 (0.05) ^aA^
Mackerel		48.83 (0.17) ^aB^	3.19 (0.10) ^aB^	64.65 (1.43) ^aB^	134.63 (4.45) ^aB^	34.45(1.14) ^aB^	2.90 (0.10) ^aB^
		57.05 (0.37) ^aC^	2.92 (0.17) ^aA^	92.24 (3.56) ^aC^	199.07 (8.19) ^aC^	44.61 (1.84)^aA^	2.24 (0.09) ^aA^
	Papain Pepsine Protamex^TM^	58.51 (0.77) ^bA^	2.17 (0.12) ^bA^	138.83 (6.14) ^bA^	256.63 (2.64) ^bA^	55.39 (0.57) ^bA^	1.81 (0.02) ^bA^
Sardine		45.26 (0.29) ^bB^	2.83 (0.18) ^bB^	90.00 (1.15) ^bB^	127.72 12.07) ^bB^	34.74 (3.28) ^bB^	2.90 (0.29) ^bB^
		52.41 (1.52) ^bC^	2.63 (0.18) ^bA^	145.41 11.81) ^bC^	252.25 (6.80) ^bC^	55.61 (1.50) ^bA^	1.80 (0.05) ^bA^

Values are expressed as mean (SD, *n* = 3). Different lower-case and upper-case letters in a column denote significant differences among hydrolysates for the raw material and enzyme factors, respectively (*p* < 0.05).

**Table 2:** F-ratio values and significance levels were obtained in the multifactor ANOVA for proteolytic and bioactive properties according to these factors: fish by-product employed as raw material (F), enzyme (E), and their interaction (F × E).

	F	E	F × E
Proteolysis analysis			
Total protein	7.83 *	410.75 ***	159.41 ***
TCA-soluble proteins	60.63 ***	9.38 **	9.37 **
Soluble peptides	82.81 ***	179.36 **	34.56 ***
Free amino groups	186.77 ***	313.40 ***	72.20 ***
Degree of Hydrolysis (DH)	63.33 ***	161.40 ***	15.09 ***
Peptide Chain Length (PCL)	17.95 **	93.38 ***	4.45 *
Antioxidant capacity			
ABTS radical scavenging activity	103.79 ***	6.31 *	12.67 **
DPPH radical scavenging activity	47.42 ***	3.06 ns	9.90 **
FRAP activity	225.36 ***	860.68 ***	18.95 ***
Potential antidiabetic activity			
α-Amylase Inhibition Assay (1 mg/mL)	-	-	-
α-Amylase Inhibition Assay (10 mg/mL)	-	-	-
α-Amylase Inhibition Assay (100 mg/mL)	287.23 ***	1716.82 ***	147.46 ***
α-Glucosidase Inhibition Assay (1 mg/mL)	0.61 ns	44.78 ***	3.92 ns
α-Glucosidase Inhibition Assay (10 mg/mL)	14.73 **	230.03 ***	6.24 *
α-Glucosidase Inhibition Assay (100 mg/mL)	4.94 *	2273.75 ***	229.86 ***

ns: not significant, * *p* < 0.05, ** *p* < 0.01, *** *p* < 0.001.

## Data Availability

Data supporting the findings of this study are available on request from the corresponding author.

## Abbreviations

The following abbreviations are used in this manuscript:

ABTS	2,2′-azino-bis(3-ethylbenzothiazoline-6-sulfonic acid) diammonium salt
BSA	Bovine Serum Albumin
DH	Degree of hydrolysis
DPPH	2,2-diphenyl-1-picrylhydrazyl
MA	Mackerel by-products hydrolysed by papain
FRAP	Ferric-Reducing Antioxidant Power
ME	Mackerel by-products hydrolysed by pepsin
MR	Mackerel by-products hydrolysed by Protamex
PCA	Principal Components Analysis
PCL	Peptide Chain Length
SA	Sardine by-products hydrolysed by papain
SE	Sardine by-products hydrolysed by pepsin
SR	Sardine by-products hydrolysed by Protamex
ROS	Reactive Oxygen Species
TCA	Trichloroacetic Acid
TE	Trolox Equivalents
TNBS	2,4,6-trinitrobenzene sulfonic acid
TPTZ	2,4,6-tri(2-pyridyl)-s-triazine
